# Strategies for data normalization and missing data imputation and consequences for potential diagnostic microRNA biomarkers in epithelial ovarian cancer

**DOI:** 10.1371/journal.pone.0282576

**Published:** 2023-05-04

**Authors:** Joanna Lopacinska-Jørgensen, Patrick H. D. Petersen, Douglas V. N. P. Oliveira, Claus K. Høgdall, Estrid V. Høgdall

**Affiliations:** 1 Department of Pathology, Herlev Hospital, University of Copenhagen, Herlev, Denmark; 2 Department of Gynaecology, Juliane Marie Centre, Rigshospitalet, University of Copenhagen, Copenhagen, Denmark; Institute of Parasitology and Biomedicine, SPAIN

## Abstract

MicroRNAs (miRNAs) are small non-coding RNA molecules regulating gene expression with diagnostic potential in different diseases, including epithelial ovarian carcinomas (EOC). As only a few studies have been published on the identification of stable endogenous miRNA in EOC, there is no consensus which miRNAs should be used aiming standardization. Currently, U6-snRNA is widely adopted as a normalization control in RT-qPCR when investigating miRNAs in EOC; despite its variable expression across cancers being reported. Therefore, our goal was to compare different missing data and normalization approaches to investigate their impact on the choice of stable endogenous controls and subsequent survival analysis while performing expression analysis of miRNAs by RT-qPCR in most frequent subtype of EOC: high-grade serous carcinoma (HGSC). 40 miRNAs were included based on their potential as stable endogenous controls or as biomarkers in EOC. Following RNA extraction from formalin-fixed paraffin embedded tissues from 63 HGSC patients, RT-qPCR was performed with a custom panel covering 40 target miRNAs and 8 controls. The raw data was analyzed by applying various strategies regarding choosing stable endogenous controls (geNorm, BestKeeper, NormFinder, the comparative ΔCt method and RefFinder), missing data (single/multiple imputation), and normalization (endogenous miRNA controls, U6-snRNA or global mean). Based on our study, we propose hsa-miR-23a-3p and hsa-miR-193a-5p, but not U6-snRNA as endogenous controls in HGSC patients. Our findings are validated in two external cohorts retrieved from the NCBI Gene Expression Omnibus database. We present that the outcome of stability analysis depends on the histological composition of the cohort, and it might suggest unique pattern of miRNA stability profiles for each subtype of EOC. Moreover, our data demonstrates the challenge of miRNA data analysis by presenting various outcomes from normalization and missing data imputation strategies on survival analysis.

## Introduction

Ovarian cancer, the eighth leading cause of cancer-related death among females worldwide [1], is a heterogenous disease with several histologic subtypes with epithelial (EOC) being most predominant (90–95% of cases) [1–3]. EOC can be further categorized into four main subtypes: serous (75%), endometrioid (10%), clear cell (10%), and mucinous (3%) that account for more than 95% of cases [2]. Its heterogeneity and lack of early screening methods accounts for the high mortality rates [3, 4].

MicroRNAs (miRNAs) are non-coding molecules that play an active role in regulation of gene expression and have been linked with many diseases, including EOC [5–7]. Despite numerous studies, there are not yet miRNA cancer biomarkers available, which inevitably leads to the main question: will it be ever feasible to implement miRNA biomarkers in the clinic? [8]. As there is a lack of consensus regarding optimal methodologies for performing miRNA detection, data analysis and standardizations, the conclusion from various miRNA studies is not consistent [9, 10]. Accurate quantification of miRNAs is a challenging task, because of their short length, high sequence similarities, occurrence of isoforms and *O*-methyl modifications [11]. Moreover, the miRNAs account only for a small fraction (ca. 0.01%) of total RNA in a sample and their expression varies from a few to thousands of copies per cell [12]. Real-time RT-qPCR is the golden standard within the field of miRNAs detection and it is commonly used to validate findings from large-scale miRNA profiling studies [13, 14]. However, RT-qPCR can be performed by various technologies that employ different strategies, e.g., stem-loop reverse transcriptase-PCR, polyadenylation of RNAs, ligation of adapters or RT with universal or miRNA-specific qPCR primers and/or probes [15]; all of which may further impact the outcome of results. Moreover, there is no consensus regarding data handling and analysis and the impact of various normalization methods has basis of discussion [16–19]. Exogenous oligonucleotides, which are added at known amount to the biological specimens during RNA isolation or cDNA synthesis, can only correct RT-qPCR data for the variability arising from particular technical steps, but not for any other variables to which they are not exposed, such as disease stage and progression, medical treatment or sample collection and preservation [17, 18]. Ideally, the normalization methods should rely on the use of stably expressed endogenous miRNAs, which potentially eliminates the differences resulting from RNA class origin and sampling, thus might help to discover unique and reproducible changes in miRNA expression levels [18]. U6, a small nuclear RNA (snRNA), is commonly used as the endogenous control in miRNA studies, despite reported high inter-variances and expression instability in cancers [20, 21]. Moreover, U6-snRNA belongs to the other class of RNA than miRNAs, and thus, their transcription, processing and tissue-specific expression patterns are different [18]. Thus, despite its exhaustive use, U6-snRNA has a series of limiting factors which might compromise the result outcome. To date, only a few reports on the identification of endogenous miRNAs in OC have been published [4, 22–24]. Another challenging step in data analysis is related to the fact that it is often not possible to collect complete data in RT-qPCR experiments. Cp values for some miRNAs can be missing because of technical failure or biological reason. Unfortunately, studies tend not to precisely describe how handling of missing data was performed, e.g., the number/ratio of missing values per variable of interest is not included or cut-off regarding acceptable amount of missingness is not presented [25, 26]. Therefore, our aim was to validate stability of previously reported endogenous miRNA controls in OC in a new cohort of patients. As differences between observed miRNAs levels in various studies have been reported, we validated our finding in two external cohorts retrieved from the NCBI Gene Expression Omnibus database. Moreover, we compared various missing data approaches (complete cases, single or multiple imputation) and data normalization strategies (endogenous miRNA controls, U6-snRNA, and global mean) in terms of their impact on the survival analysis.

## Materials and methods

### Patient cohort

Tumor tissues stored as formalin-fixed and paraffin embedded (FFPE) were acquired from two Danish projects: the Pelvic Mass study (2004–2014) and the GOVEC (Gynecological Ovarian Vulva Endometrial Cervix cancer) study (2015 –ongoing) through the Bio- and Genome Bank Denmark [27]. The study has been approved by the Danish National Committee for Research Ethics, Capital Region (H-17029749/H-15020061) and was performed according to the guidelines of the Declaration of Helsinki, including written informed consent from all patients. Tissues from a total of 63 patients diagnosed with EOC (International Federation of Gynecology and Obstetrics staging—FIGO stage III/IV) were included in this study. All patients were followed from the surgery date until either death of any cause, emigration or November 11, 2022.

### RNA extraction and miRNA profiling by RT-qPCR

Total RNA was extracted from FFPE tissue slices by use of miRNeasy FFPE kit (Qiagen, cat. No. 217504) and subjected to RT-qPCR as described previously [28]. Briefly, two tissue sections with 5 μm thickness were sliced from each paraffin block and immersed in 160 μl deparaffinization solution (Qiagen), followed by steps described by the manufacture’s protocol. 1 μl RNA isolation spike-in control mix (UniSp2, UniSp4, and UniSp5) (Qiagen) was added to each sample. Reverse transcription of RNA was accomplished by miRCURY LNA RT Kit (Qiagen, cat. no. 339340) by adding 10 ng of total RNA and 0.5 μl cDNA synthesis spike-in control mix containing UniSp6 and cel-miR-39-3p in 10 μl reaction volumes. RT-qPCR reactions were performed using miRCURY LNA SYBR Green PCR Kit (Qiagen, cat. no. 339347), miRCURY Custom PCR Panels in a 384-well plate format (Qiagen, cat. no. 339332, design no. YCA33809) and a LightCycler 480 (Roche, Hvidovre, Denmark).

The miRCURY Custom PCR panels contained 48 locked nucleic acid (LNA) PCR assays for detection of 1) 40 miRNAs selected based on their stability or biomarker potential, 2) the RNA isolation spike-in controls added at the beginning of each isolation procedure (UniSp2, UniSp4, and UniSp5), 3) the cDNA synthesis spike-in controls (UniSp6 and cel-miR-39-3p), 4) UniSp3, the interplate calibrator (UniSp3_IPC), to identify between runs variations, 5) U6-snRNA which is commonly used as the endogenous control, and 6) blank spot to control unwanted RT-qPCR outcomes. A design of a customized plate can be found in S1 Fig. RT-qPCR reactions were prepared according to the manufacture’s protocol. Briefly, a large pool containing the 2x miRCURY SYBR Green Master Mix and RNase free water was prepared, and cDNA was added at a ratio of 1:100 of a total volume. The mixture was then homogenized thoroughly and briefly centrifuged before distributing 10 μl into individual PCR wells. The LightCycler®480 software version 1.5 (Roche) and absolute quantification analysis/2^nd^ derivative maximum method with high confidence setting was employed to determine the baseline and the Crossing points (Cps) of the amplification curves for each run. The customized panels were subjected to RT-qPCR experiments and raw data was collected from 10 panels (8 patients per panel) (S2 Table). After interplate calibration with UniSp3_IPC, the isolation spike-in controls (UniSp2, UniSp4, and UniSp5) and the cDNA synthesis controls (UniSp6 and cel-miR-39-3p) were then used to evaluate the efficiency of the process and exclude any samples due to questionable quality, described according to previously published protocol [28]. The isolation spike-in controls are mixed such that UniSp2 is present at a concentration 100-fold higher than UniSp4, and UniSp4 is present at a concentration 100-fold higher than UniSp-5. Therefore, approximately 6.6 cycles difference is expected between UniSp4 and UniSp2, as well as between UniSp5 and UniSp4. As the concentration of UniSp5 reflects weakly expressed miRNAs, its detection might not always be possible. Moreover, since the input RNA amount for cDNA synthesis had to be adjusted to 10 ng in total, the cDNA samples were prepared with different RNA dilution ratios, as the concentrations of isolated RNA varied widely, which led to relatively high variation in observed UniSp2 and UniSp4 levels. Therefore, all samples with UniSp2 Cp values above 30 and outside 5–8 Cp difference range between UniSp4 and UniSp2 were excluded from further analysis (S2 Table).

### Data handling and analysis

**Fig 1:**
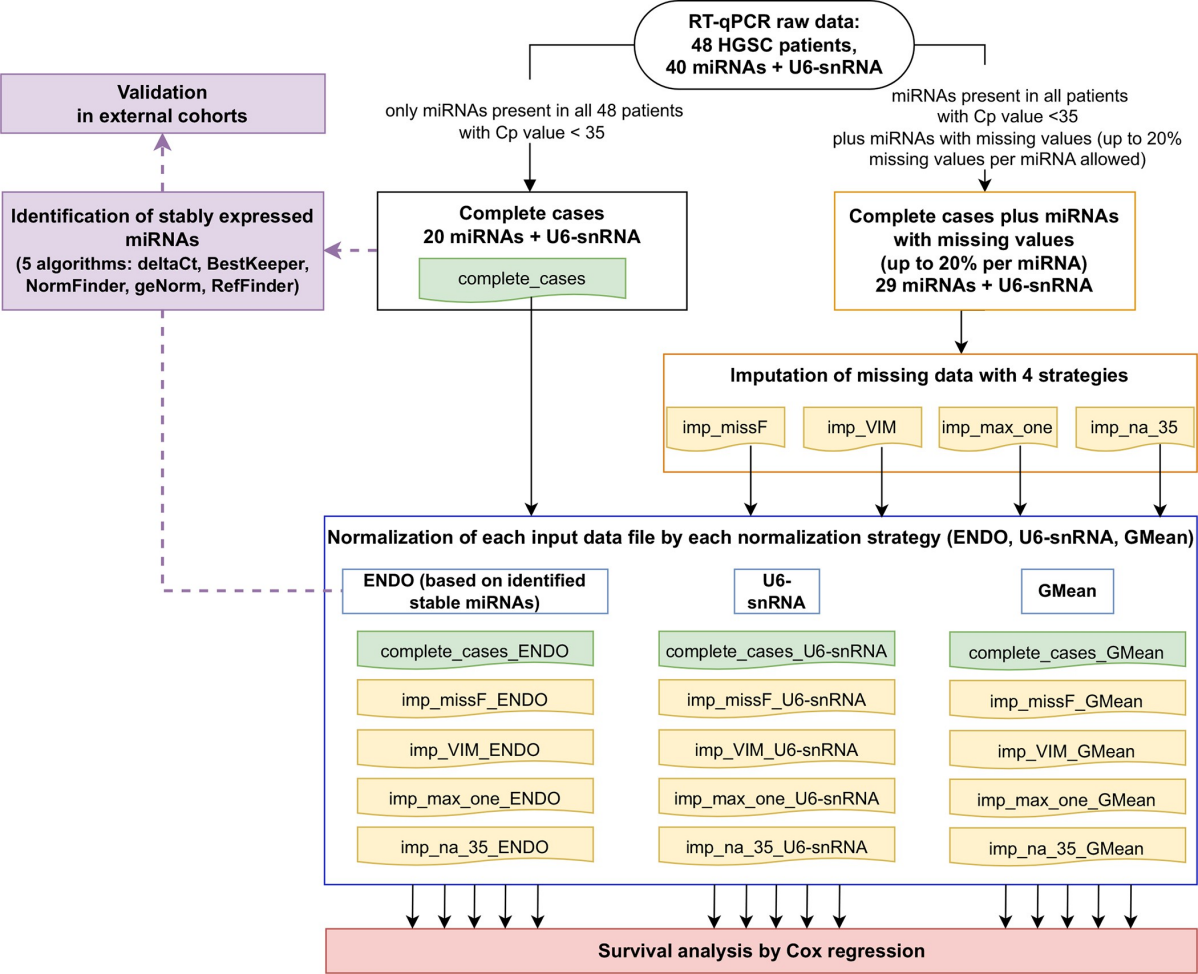
Analysis pipeline for comparison of missing data and data normalization handling strategies impact on identification of stable endogenous miRNAs controls and survival analysis. Only miRNAs without missing values (complete_cases) were included to assess their stability with five algorithms: the comparative delta-Ct method (deltaCt), BestKeeper, NormFinder, geNorm, and RefFinder. Our findings were further validated in external cohorts. Single or multiple imputation methods were applied on miRNAs with missing value ratio lower than 20%. Three normalization strategies were used individually on different input datasets. Finally, survival analysis by Cox regression was performed on fifteen differently processed datasets originated from the same RT-qPCR raw dataset.

All data analyses were performed using R Statistical Software (version 4.1.1; R Foundation for Statistical Computing, Vienna, Austria) [29]. The analysis workflow employed is presented on Fig 1.

### Endogenous control selection

In order to identify stable endogenous controls, only miRNAs without missing values and Cp values below 35 (complete cases) were included. The stability of the miRNA investigated was assessed by utilizing online expression stability tool RefFinder [30] implemented into R software by use of R package: RefSeeker developed by our group. The tool enables the identification of stably expressed candidates by several well-known algorithms; BestKeeper [31], NormFinder [32], geNorm [19] and the comparative delta-Ct method [33]. Moreover, RefFinder assigns a weight to the ranking of the four statistical algorithms and calculate a geometric mean to determine an overall ranking of the analyzed genes.

### Validation in external datasets

We retrieved data from two independent datasets: GSE81873 [34] and GSE43867 [35] by use of the NCBI Gene Expression Omnibus database. For detailed information regarding biospecimen collection, clinical data, and sample processing, we refer to the original publications for each dataset. We employed the R package miRBaseConverter to unify miRNA annotation from two datasets to the latest miRBase version (version 22) [36]. MiRNA entries not presented in the latest version of the miRBase database were excluded from further analysis. Only targets (miRNAs and controls) without missing values across entire dataset and Cp values below 35 were included to perform stability analysis as described in the section”Endogenous control selection”.

### Data normalization

Three normalization methods were applied to one complete_cases and four datasets from missing data imputation step (Fig 1):

- Based on stable endogenous miRNAs (**ENDO**): normalized Cp for each miRNA for each sample is calculated by subtracting the geometric mean of stable endogenous miRNA in that sample from the raw Cp of that sample
- Based on U6-snRNA (**U6-snRNA**): normalized Cp for each miRNA for each sample is calculated by subtracting the value of U6 in that sample from the raw Cp of that sample

- Global mean made on complete cases or imputed datasets (**Gmean**): normalized Cp for each miRNA for each sample is calculated by subtracting the mean of all miRNAs (present and imputed) from the raw Cp of that miRNA.

### Missing data

The pattern for incomplete dataset was investigated by use of two R packages: “finalfit” [37] and “ggmice” [38]. The missingness of a single dependent variable–a specific miRNA with missing values was tested against explanatory variables individually: overall survival (OS), OS status, age at diagnosis, histological subtype, and a customized panel number as multiple univariate analyses. Continuous data were compared with a Kruskal Wallis test, whereas discrete data are compared with a chi-squared test [37]. In order to identify stable endogenous controls, only miRNAs without missing values were included. To investigate possible impact of different imputation methods, Cp values equal to or above 35 were marked as missing values (“NA”). The imputation methods were further applied on miRNAs with missing value ratio lower than 20%. To fill missing values, simple and multiple imputation approaches were applied. As simple imputation methods, we tested two approaches: replacing missing values with Cp = 35 (**imp_na_35**) or replacing missing values by “highest Cp value for investigated miRNA + 1” (**imp_max_one**) [39]. To fill missing values by multiple imputation, 3 strategies were employed:

- nonparametric missing value imputation using random forest enabled by an R package “missForest” [40] (**imp_missF**)
- k-nearest neighbour imputation with an R package “VIM” [41] (**imp_VIM**).

### Survival analysis

Survival analysis was performed by use of following R packages:”finalfit” [37], “RegParallel” [42],”survival” [43], and”survminer” [44] in following datasets originated from raw data, but processed in various ways (Fig 1): “complete_cases_ENDO”, “complete_cases_U6-snRNA”, “complete_cases_GMean”, “imp_missF_ENDO”, “imp_missF_U6-snRNA”, “imp_missF_GMean”, “imp_VIM_ENDO”, “imp_VIM_U6-snRNA”, “imp_VIM_GMean”, “imp_max_one_ENDO”, “imp_max_one_U6-snRNA”, “imp_max_one_GMean”, “imp_na_35_ENDO”, “imp_na_35_U6-snRNA”, “imp_ na_35_GMean”.

OS was defined as time in months, counting from the time of diagnosis (surgery) to time of death, or last censored follow-up. To investigate potential impact of various normalization and imputation strategies on survival analysis, univariate Cox proportional hazard analysis of OS (P < 0.05) was employed to identify miRNA candidates with a potential prognostic value.

The coefficient of variation for individual miRNAs (CoV_ind) was calculated as the ratio between standard deviation and mean of the Cp value of this miRNA across all samples in 4 differently imputed datasets.

## Results

The histological subtypes were 48 high-grade serous adenocarcinomas (HGSC) with the median age of 65.6 years (31.4–88.2) and the median overall survival of 31.3 months (13.1–176.1).

To evaluate performance of nine endogenous candidates, their expression was measured by RT-qPCR along the expression of miRNAs that have been reported as potential stable candidate biomarkers in OC (Table 1).

**Table 1:** Stable endogenous control and biomarker candidates investigated in the study.

**Stable endogenous control candidates**	
hsa-miR-103a-3p [23], hsa-miR-106b-3p [22], hsa-miR-149-3p [4], hsa-miR-191-5p [23], hsa-miR-24-2-5p [22], hsa-miR-24-3p [22], hsa-miR-302d-3p [22], hsa-miR-92b-5p [22], U6-snRNA	
**Biomarker candidates**	**Characteristics**
hsa-miR-101-3p	OS^1^ in other than EOC^2^ cancers [45]
hsa-miR-1183	OS [46]
hsa-miR-1234	Resistance to chemotherapy [46]
hsa-miR-126-3p	OS [46], miRNA:mRNA signatures associated with OS [47]
hsa-miR-1301	Tumour grade [48], cisplatin resistance [49]
hsa-miR-130a	Histologic subtype, advanced FIGO^3^ stage [48]
hsa-miR-135a-3p	OS [50], progression‐free survival [50, 51]
hsa-miR-139-3p	Time to progression [46]
hsa-miR-141-3p	OS and progression-free survival [52]
hsa-miR-143-3p	OS and progression-free survival in other than EOC cancers [53, 54]
hsa-miR-146b-5p	OS [55],Tumour grade [48]
hsa-miR-193a-5p	Type I or II tumours [48]
hsa-miR-195-5p	Discriminate between benign and malignant cases [56]
hsa-miR-199a-3p	Cisplatin-resistance [57]
hsa-miR-199a-5p	Cisplatin-resistance [57]
hsa-miR-200b-3p	OS and progression-free survival [52]
hsa-miR-200c-3p	OS and progression-free survival [24, 52], discriminate between benign and malignant cases [56]
hsa-miR-205-5p	Recurrence prediction [58], Subtype discrimination [24]
hsa-miR-21-5p	Discriminate between benign and malignant cases [56]
hsa-miR-221-3p	Discriminate between benign and malignant cases [56]
hsa-miR-223-3p	MiRNA:mRNA signatures associated with OS [47]
hsa-miR-23a-3p	Progression free survival [46]
hsa-miR-23a-5p	MiRNA:mRNA signatures associated with OS [47], progression free survival [46]
hsa-miR-27a-3p	OS [59]
hsa-miR-27a-5p	MiRNA:mRNA signatures associated with OS [47]
hsa-miR-34a	Histologic subtype, tumour grade, and type I or II tumours [48]
hsa-miR-455-3p	Histologic subtype, type I or II tumours [48]
hsa-miR-486-5p	MiRNA:mRNA signatures associated with OS [47]
hsa-miR-506-3p	MiRNA:mRNA signatures associated with OS [47]
hsa-miR-595	Advanced FIGO stage[48]
hsa-miR-665	OS [47]
hsa-miR-802	Time to progression [46], progression free survival [1]

^1^OS–overall survival in months

^2^EOC–epithelial ovarian carcinoma

^3^FIGO–International Federation of Gynecology and Obstetrics staging

### Identification of endogenous miRNAs controls

Only miRNAs without missing values (complete cases, Table 2) were included to assess their stability with five well-known algorithms; BestKeeper (BestK) [31], NormFinder (NormF) [32], geNorm [19], the comparative delta-Ct method (deltaCt) [33] and RefFinder [30] (Table 3). The latter algorithm assigns a weight to the ranking of the former ones and calculates a geometric mean to determine an overall ranking of the analyzed miRNAs. The differences between rankings from various algorithms can be seen, for example hsa-miR-23a-3p is ranked as most stable by deltaCt, NormF, geNorm and RefFinder, but according to the BestK is ranked at the 8^th^ position. U6-snRNA, commonly used control is not among top most stable candidates for various algorithms.

**Table 2:** The expression values of miRNAs and controls presented as their mean (mean Cp), standard deviation (sd) and ratio of missing values (ratio NA).

Complete cases			miRNAs with missing values (up to 20% missing values)				miRNAs with > 20% missing values	
identification of stably expressed miRNAs			used together with complete cases to investigate data imputation impact				excluded from data analysis	
*Target*	*mean*	*sd*	*Target*	*Ratio NA [%]*	*mean*	*sd*	*Target*	*Ratio NA [%]*
U6-snRNA	24.35	1.33	hsa-miR-106b-3p	2.1	31.68	1.50	hsa-miR-1183	97.9
hsa-miR-101-3p	26.15	1.39	hsa-miR-1234-3p	18.8	29.51	0.96	hsa-miR-135a-3p	83.3
hsa-miR-103a-3p	25.5	1.29	hsa-miR-126-3p	16.7	26.70	1.74	hsa-miR-149-3p	39.6
hsa-miR-139-3p	32.06	0.99	hsa-miR-1301-3p	16.7	31.78	1.46	hsa-miR-23a-5p	62.5
hsa-miR-141-3p	24.16	1.81	hsa-miR-130a-3p	6.2	26.04	1.29	hsa-miR-27a-3p	29.2
hsa-miR-143-3p	25.92	1.58	hsa-miR-205-5p	4.2	27.30	2.81	hsa-miR-27a-5p	29.2
hsa-miR-146b-5p	27.31	1.51	hsa-miR-24-2-5p	8.3	31.67	1.12	hsa-miR-302d-3p	97.9
hsa-miR-191-5p	26.37	1.42	hsa-miR-24-3p	10.4	24.42	1.75	hsa-miR-506-3p	60.4
hsa-miR-193a-5p	29.28	1.09	hsa-miR-486-5p	8.3	31.54	1.70	hsa-miR-595	100.0
hsa-miR-195-5p	27.32	1.71					hsa-miR-802	100.0
hsa-miR-199a-3p	25.08	1.94					hsa-miR-92b-5p	33.3
hsa-miR-199a-5p	27.02	1.95				
hsa-miR-200b-3p	25.58	1.52				
hsa-miR-200c-3p	23.95	1.68				
hsa-miR-21-5p	21.52	1.21				
hsa-miR-221-3p	26.89	1.17				
hsa-miR-223-3p	27.27	1.49				
hsa-miR-23a-3p	24.12	1.27				
hsa-miR-34a-5p	26.83	1.11				
hsa-miR-455-3p	29.41	1.12				
hsa-miR-665	31.45	1.28				

**Table 3:** Stability values and rankings for five algorithms used to identify best stable endogenous controls. The stability values are not directly comparable between different algorithms.

	deltaCt		BestKeeper		NormFinder		geNorm		RefFinder	
**Target**	**Stability_value_deltaCt**	**Rank**	**Stability_value_BestK**	**Rank**	**Stability_value_NormF**	**Rank**	**Stability_value_NormF**	**Rank**	**Geom. mean value**	**Rank**
hsa-miR-23a-3p	1.275	1	1.052	8	0.642	1	0.855	1	1.682	1
hsa-miR-193a-5p	1.295	2	0.774	1	0.681	2	0.960	6	2.213	2
hsa-miR-21-5p	1.308	4	0.961	6	0.701	3	0.942	5	4.356	3
hsa-miR-101-3p	1.306	3	1.168	13	0.710	4	0.903	4	4.998	4
hsa-miR-221-3p	1.325	5	0.949	5	0.717	5	0.981	7	5.439	5
hsa-miR-146b-5p	1.373	8	1.266	17	0.832	8	0.855	1	5.743	6
hsa-miR-191-5p	1.325	5	1.175	14	0.735	6	0.886	3	5.958	7
hsa-miR-455-3p	1.395	9	0.914	4	0.863	9	1.032	9	7.348	8
hsa-miR-34a-5p	1.419	10	0.864	3	0.891	10	1.065	10	7.401	9
hsa-miR-103a-3p	1.356	7	1.053	9	0.781	7	0.998	8	7.707	10
hsa-miR-139-3p	1.546	13	0.809	2	1.084	13	1.172	13	8.142	11
U6-snRNA	1.688	15	1.036	7	1.287	14	1.266	15	12.186	12
hsa-miR-143-3p	1.469	11	1.326	18	1.021	11	1.104	11	12.441	13
hsa-miR-195-5p	1.521	12	1.434	19	1.081	12	1.127	12	13.461	14
hsa-miR-223-3p	1.686	14	1.216	16	1.297	15	1.217	14	14.727	15
hsa-miR-665	1.808	17	1.091	10	1.445	17	1.407	18	15.102	16
hsa-miR-200b-3p	1.757	16	1.139	11	1.430	16	1.453	19	15.209	17
hsa-miR-200c-3p	1.839	19	1.162	12	1.563	18	1.498	20	16.926	18
hsa-miR-199a-3p	1.838	18	1.582	21	1.568	19	1.323	16	18.412	19
hsa-miR-199a-5p	1.862	20	1.568	20	1.592	20	1.364	17	19.204	20
hsa-miR-141-3p	1.926	21	1.214	15	1.661	21	1.539	21	19.306	21

### Validation in external datasets

Our results were validated using two external datasets retrieved from the NCBI Gene Expression Omnibus database: GSE81873 and GSE43867 (Table 4). After filtering miRNAs with missing values and values above 35, there were 81 and 162 targets, respectively in the cohorts GSE81873 and GSE43867, included for the stability analysis. Rankings for miRNAs shared between cohorts are presented in Tables 5 and 6, whereas full rankings for both datasets can be found in the S3 Table. The differences between rankings were observed, for example hsa-miR-193a-5p was ranked among top candidates for our cohort (RefFinder rank 2/21) and GSE81873 dataset (RefFinder rank 10/89), but not for the GSE43876 cohort (RefFinder rank 72/162), when only HGSC patients were included. U6-snRNA was ranked as 12 in our dataset, 45 in GSE81873, and 161 in GSE43876 according to RefFinder stability rank. The differences between various algorithms can be seen and are more pronounced with higher number of miRNAs being included in the analysis. In order to evaluate the composition of the cohort on the choice of endogenous controls, we performed stability analysis in different subgroups in the external cohorts and presented rankings for chosen miRNAs in Table 6.

**Table 4:** Characteristics of external cohorts included in the validation study.

	study_dataset	GSE81873_dataset			GSE43867_dataset	
Cohort composition	48 HGSC patients	16 HGSC, 3 LGSC, 13 benign			66 HGSC, 20 LGSC	
Targets	40 miRNAs + U6-snRNA + controls	552 miRNAs + U6-snRNA + controls			663 miRNAs + U6-snRNA + controls	
Platform	miRCURY Custom PCR Panels (Qiagen)	TaqMan® OpenArray® Human MicroRNA Panel (miRBase v14) (Applied Biosystems/Thermo Fisher Scientific)			TaqMan Array Human microRNA A/B Cards v2.0 (Applied Biosystems/Thermo Fisher Scientific)	
Preprocessing	Unifying miRNAs names to miRBase (v22) database					
Filtering	Only targets without missing values across entire dataset and Cp values below 35 included					
Targets_filtered	21 targets (20 miRNAs + U6-snRNA)	89 targets (83 miRNAs + U6-snRNA + 5 other targets)			162 targets (160 miRNAs + U6-snRNA + 1 another target)	
Stability analysis	study_HGSC	GSE81873_HGSC	GSE81873_SC	GSE81873_SC+benign	GSE43867_HGSC	GSE43867_SC
	48 HGSC patients	16 HGSC patients	19 SC patients	32 patients	66 HGSC patients	86 SC patients
	Table 3					
	Table 5				Table 5	
		Table 6				

**Table 5:** Comparison of stability rankings for shared miRNAs between our cohort and two external datasets: GSE81873 and GSE43867.

	study_subset					GSE81873_HGSC					GSE43867_HGSC				
	**48 HGSC patients**					**16 HGSC patients**					**66 HGSC patients**				
	**21 targets**					**89 targets**					**162 targets**				
**Target**	deltaCt	BestKeeper	NormFinder	geNorm	**RefFinder**	deltaCt	BestKeeper	NormFinder	geNorm	**RefFinder**	deltaCt	BestKeeper	NormFinder	geNorm	**RefFinder**
**hsa-miR-193a-5p**	2	1	2	6	**2**	7	40	6	15	**10**	136	5	137	139	**72**
**hsa-miR-221-3p**	5	5	5	7	**5**	25	37	25	40	**31**	20	50	17	38	**24**
**hsa-miR-146b-5p**	8	17	8	1	**6**	36	6	36	36	**23**	44	84	42	29	**51**
**hsa-miR-191-5p**	5	14	6	3	**7**	17	41	16	10	**18**	36	100	38	16	**37**
**hsa-miR-34a-5p**	10	3	10	10	**9**	49	53	44	52	**58**	115	14	113	122	**84**
**hsa-miR-103a-3p**	7	9	7	8	**10**	3	20	3	7	**3**	46	119	61	4	**31**
**U6-snRNA**	15	7	14	15	**12**	41	83	43	16	**45**	161	162	161	161	**161**
**hsa-miR-143-3p**	11	18	11	11	**13**	34	57	32	42	**48**	123	111	123	128	**135**
**hsa-miR-195-5p**	12	19	12	12	**14**	66	76	66	62	**75**	95	146	99	66	**116**
**hsa-miR-223-3p**	14	16	15	14	**15**	74	82	74	76	**81**	119	132	121	116	**136**
**hsa-miR-199a-3p**	18	21	19	16	**19**	67	55	67	67	**73**	134	159	135	131	**152**

**Table 6:** Comparison of stability rankings for selected miRNAs in different subsets of two external datasets: GSE81873 and GSE43867.

Target	deltaCt	BestKeeper	NormFinder	geNorm	RefFinder	Dataset
**hsa-miR-191-5p**	8	29	7	5	**8**	GSE81873_SC+benign
	18	50	18	13	**21**	GSE81873_SC
	17	41	16	10	**18**	GSE81873_HGSC
	36	100	38	16	**37**	GSE43867_SC
	37	100	40	13	**38**	GSE43867_HGSC
**hsa-miR-193a-5p**	16	35	16	23	**21**	GSE81873_SC+benign
	13	42	10	19	**15**	GSE81873_SC
	7	40	6	15	**10**	GSE81873_HGSC
	136	5	137	139	**72**	GSE43867_SC
	120	5	121	126	**62**	GSE43867_HGSC
**hsa-miR-221-3p**	22	30	22	27	**26**	GSE81873_SC+benign
	29	32	29	39	**36**	GSE81873_SC
	25	37	25	40	**31**	GSE81873_HGSC
	20	50	17	38	**24**	GSE43867_SC
	22	52	18	24	**25**	GSE43867_HGSC

### Imputation of missing data

After adjusting the raw RT-qPCR data from 10 panels (8 patients per panel) by the interplate calibrator, the pattern of missingness of an incomplete dataset was investigated (Table 2). The imputation methods were further applied on miRNAs with missing value ratio lower than 20%, resulting in excluding hsa-miR-1183, hsa-miR-135a-3p, hsa-miR-149-3p, hsa-miR-23a-5p, hsa-miR-27a-3p, hsa-miR-27a-5p, hsa-miR-302d-3p, hsa-miR-506-3p, hsa-miR-595, hsa-miR-802 and hsa-miR-92b-5p. In the next step, the missingness pattern of a specific miRNA with missing values was tested against explanatory variables: OS, OS status (dead or alive), age at diagnosis, and a customized panel number, but no dependence has been observed.

In order to fill missing values, simple (na_max_one and na_35) and multiple imputation (missF, VIM) approaches were applied and their effects on the CoV values on individual miRNAs are presented in Table 7. The results show that the CoV values for individual miRNAs are not affected by multiple imputation methods (missF and VIM), however single imputation methods lead to higher CoVs when comparing to CoVs from the raw dataset (data_raw). For hsa-miR-126-3p and hsa-miR-1234-3p, which had many missing values, the CoVs are approximately 1.6 times higher than CoVs from the raw dataset.

**Table 7:** Coefficient of variation analysis on individual miRNAs that presented missing Cp values and were imputed with four methods (missF, VIM, na_max_one, na_35). CoV, CoV_imp_missF, CoV_imp_VIM, CoV_imp_max_one, and CoV_imp_na_35 –coefficient of variation for particular RNA in a raw dataset, in the missF imputed dataset, in the VIM imputed dataset, in max_one imputed dataset and na_35 imputed dataset, respectively.

Target	RatioNA [%]	CoV	CoV_imp_missF	CoV_imp_VIM	CoV_imp_max_one	CoV_imp_na_35
**hsa-miR-106b-3p**	2,1	4,7	4,7	4,8	5	4,9
**hsa-miR-1234-3p**	18,8	3,3	3	3	5,3	7,6
**hsa-miR-126-3p**	16,7	6,5	6,2	6,3	10,4	12,5
**hsa-miR-1301-3p**	16,7	4,6	4,4	4,4	6	5,6
**hsa-miR-130a-3p**	6,2	5	4,9	4,8	6,2	9,5
**hsa-miR-205-5p**	4,2	10,3	10,1	10,1	11,4	11,4
**hsa-miR-24-2-5p**	8,3	3,5	3,4	3,5	4,2	4,4
**hsa-miR-24-3p**	10,4	7,2	6,8	6,9	12,2	14,3
**hsa-miR-486-5p**	8,3	5,4	5,2	5,2	6	5,9

In the next step, three different methods of normalization (ENDO, U6-snRNA, GMean) were performed on complete cases dataset and on each imputed dataset, which resulted in fifteen different datasets. Each miRNA was subjected to univariate Cox regression analyses of OS and miRNA candidate OS predictors were found only in datasets that were imputed, but not in complete cases dataset normalized by three methods (ENDO, U6-snRNA and GMean) (Table 8). Hsa-miR-126-3p was indicated as associated with OS in two workflows: imp_na_35_U6-snRNA and imp_max_one_U6-snRNA, whereas hsa-miR-1301-3p was found in: imp_VIM_GMean, imp_missF_GMean, imp_na_35_U6-snRNA, imp_max_one_U6-snRNA, imp_VIM_U6-snRNA, imp_na_35_GMean, and imp_missF_U6-snRNA. Noticeably, both miRNAs: hsa-miR-126-3p and hsa-miR-1301-3p had missing values in a raw dataset (16.7% of missing values, Table 7).

**Table 8:** Univariate Cox analyses to identify the candidate predictors for OS. miRNAs with P-value below 0.05 are presented.

Variable	P	LogRank	HR	HRlower	HRupper	Workflow
hsa-miR-126-3p	0.0379	0.0339	0.8027	0.6523	0.9878	imp_na_35_U6-snRNA
hsa-miR-126-3p	0.0393	0.0372	0.7959	0.6406	0.9889	imp_max_one_U6-snRNA
hsa-miR-1301-3p	0.0086	0.0076	0.4362	0.235	0.8098	imp_VIM_GMean
hsa-miR-1301-3p	0.0138	0.0133	0.4505	0.2389	0.8497	imp_missF_GMean
hsa-miR-1301-3p	0.0303	0.027	0.6982	0.5044	0.9664	imp_na_35_U6-snRNA
hsa-miR-1301-3p	0.0354	0.0325	0.7138	0.5214	0.9773	imp_max_one_U6-snRNA
hsa-miR-1301-3p	0.0361	0.0321	0.6859	0.4821	0.9759	imp_VIM_U6-snRNA
hsa-miR-1301-3p	0.0395	0.0397	0.5915	0.3589	0.975	imp_na_35_GMean
hsa-miR-1301-3p	0.0495	0.0452	0.7041	0.4961	0.9993	imp_missF_U6-snRNA

## Discussion

With this study, hsa-miR-23a-3p, and hsa-miR-193a-5p were identified as most stable in a cohort of 48 patients with HGSC. Interestingly, these miRNAs were included in our study because they were reported previously as potential biomarkers in EOC (Table 1) [60] to discriminate between type I and II tumours (hsa-miR-193a-5p) and to predict progression free survival (hsa-miR-23a-3p) pointing towards an explanation of the very different effects of miRNA observed in cohorts with different composition of EOC patients. Moreover, as we showed in Table 6, the pattern of stability for a particular miRNA (increased or decreased stability rank) is dependent on the histological composition of the cohort, but also varies between different miRNAs. For example, hsa-miR-193a-5p stability is increasing, when there is only one subtype of EOC present (HGSC) versus a cohort with SC and benign cases included (GSE81873), however, opposite effect is observed for hsa-miR-191-5p. In the study that used the cohort GSE43867, two miRNAs were used as endogenous stable controls: hsa-miR-16-5p and hsa-miR-191-5p, however how they were chosen is not precisely described. According to our stability analysis, these miRNAs were not among top ten most stable targets for any of the algorithms and according to RefFinder they were ranked on the position 37 –hsa-miR-191-5p and on the position 121 –hsa-miR-16-5p (S3 Table). In case of GSE81873, the top 10 microRNAs obtained from the geNorm algorithm were used to normalize data. However, the names of these miRNAs are not mentioned in the description, therefore it would be difficult to compare it with our analysis. Nevertheless, different stability algorithms selected various top 10 miRNAs for this dataset according to our stability analysis (S3 Table). Therefore, in general more detailed description of various data processing steps would be recommended to enable validation of the results across different research centers.

U6-snRNA that is commonly used as the endogenous control in OC studies [61–63] was not in the top 10 of most stable candidates for three algorithms: deltaCt, NormFinder, geNorm, and RefFinder (rank 14 or 15 dependently on the imputation method–Table 3). Similar findings were made in external validation cohorts while considering HGSC patients, in which U6-snRNA was ranked as 45^th^ among 81 targets in GSE81873, whereas in GSE43867 was on the position 161 among 162 targets considered (Table 5). These observations indicate that U6-snRNA is not a suitable endogenous candidate for normalization in EOC, which is in line with some previous reports showing high inter-individual variances and expression instability of U6-snRNA in cancers [20, 21]. Our results emphasize that it is crucial to select suitable endogenous controls to ensure the reliability of RT-qPCR, especially when working with miRNAs in a clinical context in a complex, heterogenous diseases such as EOC, as the conclusions that are drawn might be dependent on the composition of the cohort and might impact clinical decisions.

In our previous study, we collected miRNA-microarray data from four datasets: the in-house “Pelvic Mass”, and three public datasets with primary EOC patients: The Cancer Genome Atlas, GSE47841, and GSE73581 in order to find endogenous control candidates [22]. We found that two miRNAs: hsa-miR-106b-3p and hsa-miR-92b-5p were among the top 100 candidates for all datasets, when considering only miRNAs mutual for all datasets. Their stability was not evaluated in this study, as none of them were available as a complete case (hsa-miR-106b-3p – 2.1% of missing data, hsa-miR-92b-5p – 33.3% of missing data).

Moreover, we furthermore examined the impact of various data handling on survival analysis in 48 HGSC patients. Each miRNA in each of 15 differently processed datasets (Fig 1) was subjected to univariate Cox regression analysis and miRNA being predictors of OS (hsa-miR-126-3p and hsa-miR-1301-3p) were only found in the datasets that included miRNAs with missing values (Table 8). Interestingly, these miRNAs were included because of their biomarker potential: hsa-miR-126-3p was associated with OS in EOC and hsa-miR-1301 was shown to be involved in cisplatin resistance (Table 1). However, these miRNAs were not complete cases (Table 2) and further investigation is required to understand their role in EOC. As shown in Table 7, the CoV values for individual miRNAs were mainly affected by single imputation methods, whereas subtle differences were observed when comparing datasets filled by multiple imputation methods with the raw dataset. This is not surprising, as imputed values are estimated from the detected Cp values. However, such imputation might assign Cp values to some miRNAs that were in fact not detectable because of a biological reason (no target in the sample or very low concentration) not a technical failure and might lead to false conclusions [39]. Therefore, one might consider increasing the number of biological/technical replicates per sample in order to decrease the number of miRNAs with missing data or to confirm their missingness because of biological reasons, such as regulatory loops of other genes expression depending on the biological context of each EOC subtype. Moreover, the sample age and type could be additionally included as a potential factor that could impact miRNA studies. Nonetheless, previous studies have showed a good correlation of miRNA between fresh-frozen and FFPE when including several hundred microRNAs [64, 65].

## Conclusions

Our study demonstrates the need of awareness in the choice of RT-qPCR missing data handling and data normalization approaches in miRNA biomarker studies. We suggest that other endogenous controls than U6-snRNA, which seems to be unstable in various EOC cohorts, should be considered and we identified hsa-miR-23a-3p and hsa-miR-193a-5p among top candidates for HGSC patients. We presented that the pattern of miRNA stability depends on the histological composition of the cohort and it might suggest that each subtype of EOC might be characterized by its unique miRNAs expression pattern. Moreover, in order to achieve consensus on miRNA-related studies in EOC, future studies regarding miRNA data analysis are required, as e.g., many studies do not precisely describe how handling of missing data was performed, which can lead to biased results and hinder the validation efforts.

## Supporting information

S1 Fig The customized design of 48 assays per sample in a 384 well plate format (8 samples in total). (DOCX)

S1 Table The Cp values obtained by miRNA profiling with use of the miRCURY customized PCR panels. (XLSX)

S2 Table The Cp values of spike-ins controls: UniSp2, UniSp4, UniSp5, UniSp6 and cel_miR_39_3p. (XLSX)

S3 Table Stability rankings from five algorithms (the comparative delta-Ct method, BestKeeper, Normfinder, geNorm and RefFinder) for two external datasets: GSE81873 and GSE43867. (XLSX)
