# Supplementary material for: Strategies for data normalization and missing data imputation and consequences for potential diagnostic microRNA biomarkers in epithelial ovarian cancer
Source: PLoS One. 2023 May 4;18(5):e0282576. doi: 10.1371/journal.pone.0282576 (PMC10159121; doi:10.1371/journal.pone.0282576)
Supplement: S1 Fig — (DOCX) [file pone.0282576.s001.docx]

**S1 Fig. The customized design of 48 assays per sample in a 384 well plate format (8 samples in total)**
